# Cancer care coordination in rural Hawaii: a focus group study

**DOI:** 10.1186/s12913-024-10916-1

**Published:** 2024-04-24

**Authors:** Shin Chang, Michelle Liu, Christa Braun-Inglis, Randall Holcombe, Izumi Okado

**Affiliations:** 1 John A Burns School of Medicine, University of Hawaiʻi at Mānoa, 651 Ilalo St, 96813 Honolulu, HI USA; 2 University of Hawai‘i Cancer Center, 701 Ilalo St. 6th Floor, 96813 Honolulu, HI USA; 3 University of Vermont Cancer Center, 149 Beaumont Av. Burlington, 05405 VT, USA

**Keywords:** Cancer, Care coordination, Qualitative study, Focus group, Rural Hawaii

## Abstract

**Background:**

Rural populations consistently experience a disproportionate burden of cancer, including higher incidence and mortality rates, compared to the urban populations. Factors that are thought to contribute to these disparities include limited or lack of access to care and challenges with care coordination (CC). In Hawaii, many patients residing in rural areas experience unique challenges with CC as they require inter-island travel for their cancer treatment. In this focus group study, we explored the specific challenges and positive experiences that impact the CC in rural Hawaii cancer patients.

**Methods:**

We conducted two semi-structured focus group interviews with cancer patients receiving active treatment for any type of cancer (*n* = 8). The participants were recruited from the rural areas of Hawaii, specifically the Hawaii county and Kauai. Rural was defined using the Rural-Urban Commuting Area Codes (RUCA; rural ≥ 4). The focus group discussions were facilitated using open-ended questions to explore patients’ experiences with CC.

**Results:**

Content analysis revealed that 47% of the discussions were related to CC-related challenges, including access to care (27.3%), insurance (9.1%), inter-island travel (6.1%), and medical literacy (4.5%). Other major themes from the discussions focused on facilitators of CC (30.3%), including the use of electronic patient portal (12.1%), team-based approach (9.1%), family caregiver support (4.5%), and local clinic staff (4.5%).

**Conclusion:**

Our findings indicate that there are notable challenges in rural patients’ experiences regarding their cancer care coordination. Specific factors such as the lack of oncologist and oncology services, fragmented system, and the lack of local general medical providers contribute to problems with access to care. However, there are also positive factors found through the help of facilitators of CC, notability the use of electronic patient portal, team-based approach, family caregiver support, and local clinic staff. These findings highlight potential targets of interventions to improve cancer care delivery for rural patients.

**Trial Registration:**

Not required.

**Supplementary Information:**

The online version contains supplementary material available at 10.1186/s12913-024-10916-1.

## Background

Rural populations have higher cancer mortality rates compared to urban populations [1, 2]. Rural-urban disparities in cancer health outcomes are partly attributed to challenges with cancer care delivery, specifically care coordination (CC) in rural areas [1, 3]. Cancer CC is essential to high-quality cancer care [4], and since cancer CC in rural areas is affected by challenges such as limited access to providers and health services [5], it is important to gain further understanding of rural patients’ perceptions of cancer CC.

There are notable CC challenges in rural areas including limited access to specialty medical services and long travel distances to care. According to a 2022 data published by the American Society of Clinical Oncology (ASCO), only 10.5% of active oncologists practiced in rural areas, making oncologist shortage an obstacle to care access for rural cancer patients. Beyond challenges in accessing oncologists, there are other barriers to access to cancer care including the scarcity of hospitals in rural areas, limited access to health information sources, and longer travel distances needed to access health services [6–8]. There are also other notable disadvantages faced by rural populations compared with urban populations. Studies have shown that rural populations have higher poverty and unemployment rates, a greater number of uninsured residents, and lower educational attainment and health literacy [9–11]. These disadvantages, along with the lack care of access, may contribute to the higher cancer mortality rates found in rural populations.

Limited prior studies have compared the differences in cancer CC between rural and urban populations [5, 12–16]. Some studies reported more challenges with CC in rural communities compared with the urban due to the limited access to health care providers and community services, fewer providers of specialized care, and lack of effective communication between specialist and primary care [5, 13]. In contrast, studies by Mollica et al. reported more positive perceptions of CC among rural patients getting care quickly compared to urban patients [14, 15]. Some potential explanations regarding their results of more positive CC experience among rural patients include different expectations to care access in rural residents compared to urban residents, longer wait times for urban residents, and selection bias in Medicare beneficiaries. Overall, research regarding cancer CC in rural populations is limited, and there are major gaps in understanding the perceptions of CC among rural cancer patients.

Our study addresses the knowledge gap of cancer CC in rural patients, specifically in rural Hawaii, by exploring the perceptions of CC through a qualitative study. Rural Hawaii patients face challenges in seeking care such as physician shortages [17] and lack of specialty care in local clinics. Assessments on the Hawaii physician workforce prior to the COVID-19 pandemic showed that there was a high percentage of physician shortage in Hawaii, especially on the islands that are mostly rural [18]. Moreover, recent reports also estimated that 10% of providers have retired or closed their practices since the start of the pandemic [17]. With locum physicians providing oncology care in many rural areas in Hawaii, the limited medical resources and specialty cancer care in rural Hawaii mean that many cancer patients residing on the more rural islands have to travel by air to the more urban Oahu island to obtain cancer treatment. The lack of available health professionals and need for air-travel present as some of the unique barriers for rural patients in Hawaii. However, there is no prior research on how these barriers are perceived by these patients.

In this qualitative study, we explored the specific challenges and positive experiences that impact the CC in rural Hawaii cancer patients. Rural populations in Hawaii are often excluded in US population-based studies, and this study contributes new knowledge in an understudied population. These findings can be used to further improve CC in rural areas in the future.

## Methods

### Participants

In this study, we conducted two semi-structured focus group interviews with a subset of patients (*n* = 8) from a previous care coordination survey study. The care coordination study has been described elsewhere [19]. Participants were recruited from rural areas of Hawaii, specifically the Hawaii county and Kauai, and were receiving active treatment for any type of cancer. Rural was defined using the Rural-Urban Commuting Area Codes (RUCA; rural ≥ 4). The focus group participants were identified through a supplemental questionnaire in the previous study that probed for interest in focus group participation. The study was approved by the University of Hawaii Institutional Review Board.

### Study design

The research team coordinated two focus groups (*n* = 4 each) based on the times and days convenient to the participants. Prior to the start of the focus group discussions, verbal consent was again obtained from all participants. The interviews were conducted virtually over zoom (R.F.H., C.B., and I.O.) due to the pandemic and to allow remote participation. The interview questions were developed by the research team prior to focus groups and encompassed open-ended questions designed to explore care coordination (see supplement for the discussion guide). All focus group discussions were audiotaped and transcribed verbatim, with each participant’s comment anonymously attributed.

### Qualitative analysis

Analysis of the focus group interviews was conducted using content analysis. First, the researchers independently coded the transcripts to identify important aspects across the two focus group discussions. Next, the team compared, analyzed, and refined the codes until the main themes were identified. The themes were discussed until a consensus was reached. Subthemes were also identified, with the hierarchy determined and named appropriately. Any discrepancies were resolved by discussion (S.C., M.L., and I.O.).

## Results

Demographic characteristics of the participants are presented in Table 1. A majority of the participants were female (87.5%) and the mean age was 62.3. The participants were racially diverse, with 62.5% any Asian, 37.5% any White, 12.5% any Native Hawaiian, and 12.5% others. Half of the participants were breast cancer patients, while the remaining half consisted of GI and other cancer patients. There was representation across all cancer stages, with 75% of the participants in the early stages and 25% in Stage IV.

**Table 1:** Demographic characteristics of the focus group participants (*N* = 8)

Characteristics	% (*n* = 8)
Female	87.5
Age, years (mean, SD)	62.3 (11.1)
Race	
Any Asian	62.5
Any White	37.5
Any Native Hawaiian	12.5
Others	12.5
Cancer type	
Breast	50.0
GI	25.0
Other	25.0
Cancer stage	
I-III	75
IV	25.0

*Note* Because some participants were multiracial, the race data sums to more than 100%. Other cancer types include blood and urinary cancers

**Fig. 1:**
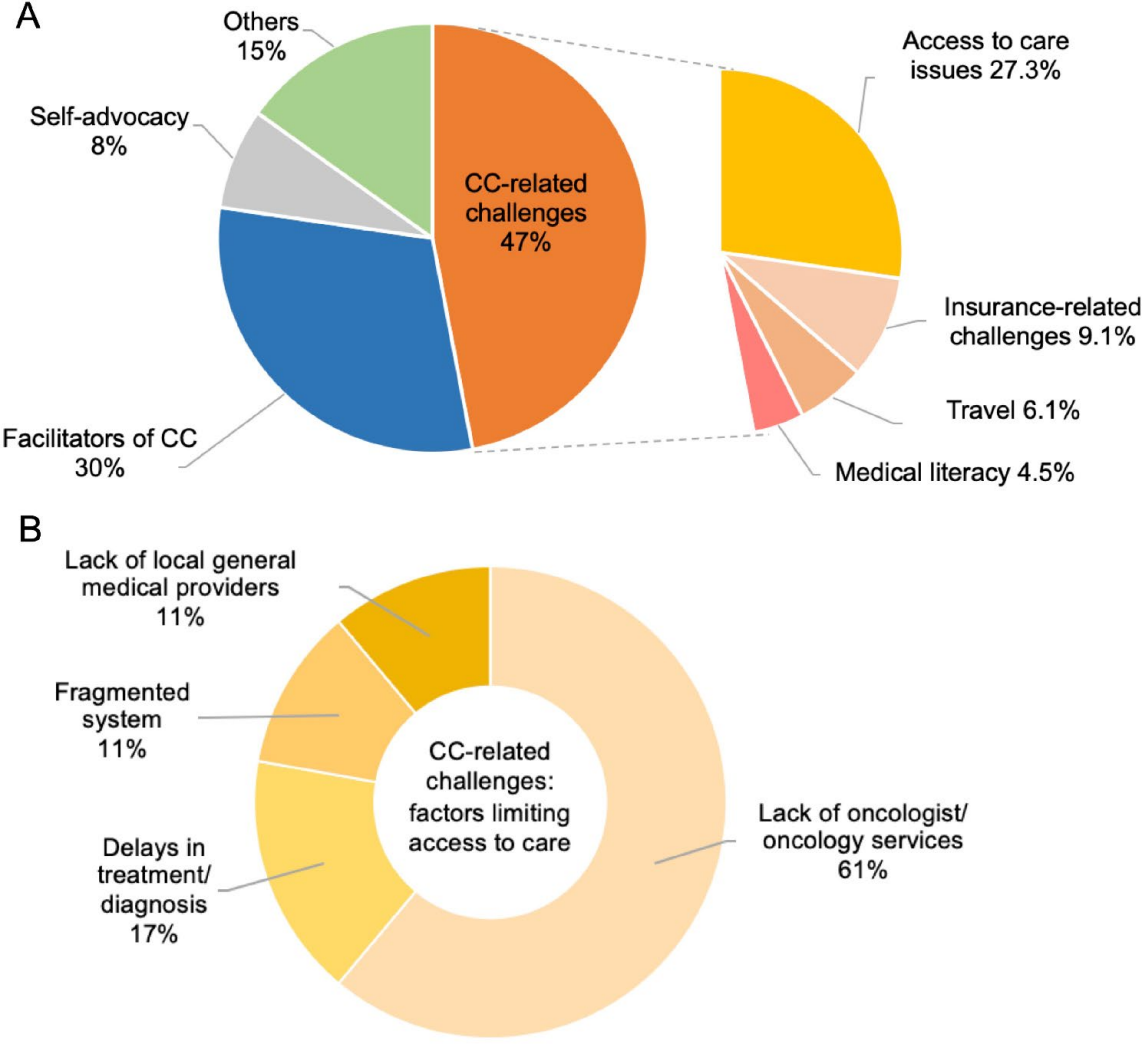
A & B Themes and subthemes, shown by proportions of focus group discussion

The content analysis (Fig. 1) revealed that almost half (47.0%) of the discussions were connected to CC-related challenges. Within CC-related challenges, access to care was the most common subtheme (27.3%), with the lack of oncologists and oncology services identified as the major contributing factor (61.1%). Multiple participants described having to be seen by several different oncologists throughout their treatment, almost to the point of having a “revolving door” of physicians (See Table 2). They mentioned that it was difficult having to be treated by physicians who did not know them well, especially because every physician has a different process and approach to care. Other contributing factors that limited access to care included delays in treatment and diagnosis (16.7%), the fragmented system (11.1%), for which patients had to undergo care across multiple healthcare organizations, and the lack of local general medical providers (11.1%). The participants discussed how the long wait times for appointments, different provider networks, and difficulty accessing local general medical providers contributed to their worries about cancer progression.

Besides access to care, other subthemes within CC-related challenges included insurance (9.1%), travel (6.1%), and medical literacy (4.5%). Some participants described issues with getting insurance-approval for treatment and inter-island travel, which either caused delays in treatment or led to high out-of-pocket costs. For other participants, the inter-island travel was difficult beyond its costs due to troubles coordinating appointment times and the time commitment required. Medical literacy may also have been a challenge, as some patients discussed their confusion regarding the roles of oncologists. These participants commented that it was strange that they were mostly visiting their oncologists rather than their primary care providers (PCPs), and questioned why their PCPs were not more involved in their cancer care.

Facilitators of CC was another major theme (30.3%) from the focus group discussions. The use of electronic patient portal (12.1%) was helpful for the 3 participants who had access to it. These participants highlighted that the portal allowed them to keep track of their health records and more easily communicate with the healthcare team. In another part of the discussion, team-based approach (9.1%) seemed to have led to a strongly positive cancer treatment experience. Other facilitators of CC included family caregiver support (4.5%), which involved patients having family members to help communicate with the care team, and local clinic staff (4.5%), especially the regular staff that are part of the clinics and infusion centers. Participants described these local staff as members of the care team who are “constant” in their care journeys and who became like family members to them.

At the end of each focus group discussion, the research team asked the participants about suggestions or advice for improving care coordination. Most participants emphasized the need for more full-time, permanent oncologists that can serve on the islands, specifically in rural areas. Besides the challenges of having a different new oncologist for nearly every visit, they mentioned that it was difficult to be kept in the dark as to why the oncologists kept leaving. They thought that it would be helpful if the medical systems were more transparent regarding physician changes, so that they could identify the local physicians with whom they could work with longer. Multiple participants also commented about the importance of self-advocacy, highlighting it as an essential component that improved and sped-up their treatment process. They described self-advocacy as asking questions, proactively reaching out to the providers for help, and keeping the care team updated, which together helped to reduce delays to care and improve their outcomes.

The remaining discussion contained other themes (15.1%). Some participants shared personal history (12.1%) regarding their cancer diagnoses, symptoms, and treatment process. Others described COVID-19 related challenges (3.0%), such as feeling isolated during COVID and having to travel under COVID-restrictions. Quotes from the focus group participants are shown in Table 2, supporting the qualitative analysis.

**Table 2:** Exemplar quotes from the focus group participants

**CC-related challenges**	
Access to care	*“… having your doctor change all the time and you don’t know them and they don’t know you and every doctor has their different process… “*
	*“…but for us here specifically on [island], the revolving door of oncologists that we have all been seeing has been a little problematic.”*
Insurance-related challenges	*“…[insurance] initially rejected giving me the [medication], so I had to stay an additional 24 hours in Honolulu to get the shot until [insurance] finally approved giving me the [medication] so I could fly home earlier, but it’s costing me thousands of dollars… “*
Travel	*“…[Insurance] won’t reimburse for the travel, or the hotel, and my husband comes with me every time…”*
Medical literacy	*“…we were informed by his PCP that he has cancer. And then after that the PCP gave us the referral to see the oncology at [Medical Center] then that’s it. So after that, she’s out of the picture…”*
**Facilitators of CC**	
Electronic Patient portal	*“[the electronic patient portal] has been awesome, it’s allowed everyone that’s been a part of my care for me to like access things, to see everything… “*
Team-based approach	*“… I am a [HMO] patient, so all my experiences are positive because they flew me over to Oahu to meet with the MDT, which is the Multidisciplinary Team. I was put in a room and all the doctors went there to talk to me.”*
Family caregiver support	*“…my dad doesn’t speak English at all–so each time, I need to go with him…if there was any problem we can always call in…”*
Local clinic staff	*“…What’s also been helpful has been the consistency, and I would say my infusion team has been amazing on the [island]. They are everyday heroes, I would never do that job, but every single one of them in the entire department I love! “*
**Self-advocacy**	
Self-advocacy	*“…I pretty much stay on top of doctors and remind them when they come in exactly where I’m at, exactly what issues I’m having with my labs, those kinds of things…”*
**Others**	
COVID-related challenges	*“…in the middle of COVID, missing that companionship during infusion and just having to be by yourself for 3, 4 hours of treatment, I think that was the part that sucked the most. “*

## Discussion

In this focus group study, we explored the specific challenges and positive experiences that impact the CC in rural Hawaii cancer patients. Overall, our findings illuminate challenges such as limited access to care, inter-island travel, and insurance-related issues as described by rural cancer patients regarding their CC. Our findings also suggest that there are facilitators of CC such as electronic patient portals, family caregiver support, and local clinic staff, along with the use of a team-based approach, that contribute to more positive experiences that may help to mitigate some of these challenges.

Our results demonstrate that access to care was a major component of CC-related challenges, with the lack of oncologists and oncology services found as the most frequently mentioned contributing factor. These findings are consistent with previous research that reported fewer practicing oncologists and general medical providers in rural areas [20–22], highlighting the need for more permanent oncologists and providers in rural areas. It is also interesting to note that the high turnover of oncologists was mentioned by the participants. Similar to suggestions made by the participants, further studies are needed to illuminate and address the factors that contribute to oncologist shortages in rural Hawaii.

Other access-related challenges included the lack of local general medical providers and the fragmented care system. Given that previous studies indicate that primary care physicians play a vital role in cancer CC by managing comorbid conditions [23], the pandemic is likely to have further exacerbated the high-turnover and challenges with finding general medical providers felt by the participants. Furthermore, other studies have also shown that fragmented cancer care results in longer time to treatment and increased mortality while potentiating existing socioeconomic disparities [24, 25]. Some of the participants were worried about these delays because they feared further cancer progression. Their worries are not unwarranted, as previous studies have shown a significant association between cancer care delays and increased mortality [26].

In addition to limited access to care, other subthemes found under CC-related challenges include travel and insurance-related issues. Many participants faced limited cancer care on their islands, were not able to get the care they required, and therefore had to travel inter-island to access the specialized treatment they needed. However, inter-island travel presented with its own unique challenges. For participants whose insurance did not cover the travel costs, the costs for travel were significant, as they needed to pay out of pocket for airfare, and air travel is the only mode of inter-island travel in Hawaii. Utilizing inter-island travels for care also meant extended time commitments and time off from work for those who were employed. Overall, these travel challenges likely further increased barriers to cancer care for rural patients. These results were supported in prior research that showed the association of increased travel burden with decreased care access for lung, breast, and colon cancer patients [27–29]. Travel distance is also associated with increased financial barriers, especially for those with lower socioeconomic status and inadequate insurance coverage in the rural population [30, 31]. Altogether, the longer travel distances faced by rural patients present as a critical health barrier for rural patients. Beyond the financial difficulties, it is important to note that cancer treatment costs can often lead to catastrophic financial burden on the patients, regardless of rural and urban residence, causing what is often termed “financial toxicity” [30, 31]. Further interventions are needed to address these financial disparities faced by cancer patients.

Lastly, patients’ lack of understanding of CC processes and the people involved in oncology care was also found as a source of challenge. Some participants did not understand why they were referred to oncologists rather than continuing care with their general providers. Further communication and explanation from the general providers about the roles of oncologists could have alleviated these confusions for patients and ensured a smoother transition in their care.

Although many specific challenges hinder rural cancer patients’ experiences with CC, our findings also revealed the strong positive impact of facilitators on patients’ CC experience. First, the participants who had access to electronic patient portals found that they could easily access their health information and communicate with care team members. By asking questions and keeping their care team up to date through the portals, they were able to better self-advocate. This suggests that electronic patient portals may help to mitigate some of the challenges caused by the long distance between rural cancer patients and their cancer care provider. However, limitations exist because the portals were not available to all our participants due to the differing locations they were at. Similarly, not all of the clinics have these systems.

Second, our findings suggest that team-based approach seemed to have led to a positive experience. It is imperative to note, however, that the comments about the team-based approach came from only one participant, the only one who was receiving an HMO-based care. Although this result may not by generalizable, a systemic review showed that team-based care may improve patient satisfaction [32]. Third, the presence of local clinic staff also contributed to positive cancer care experiences for the participants. Given the “revolving door” of oncologists, the responses given by the participants suggest that having stable and long-term relationships with the local clinic staff made participants feel like they have constant team members who will keep being a part of their cancer journeys. This is in strong contrast with the long list of oncologists they’ve seen, most of whom they can barely remember the name of. Finally, consistent with prior research, family caregivers’ support also helped improve the cancer CC experience for patients [33]. This is likely because family caregivers can help to alleviate the emotional toll of cancer on patients while also helping to manage the health care needs and CC-related tasks for patients.

There are some limitations to this study. Firstly, the participants for the focus groups were selected based on voluntary participation. It cannot be ruled out that positive or negative experiences may have prompted these participants to be more willing to participate in this study. Secondly, this study is composed of two focus groups, with an overall sample size of 8 participants. It should be noted that the study was originally composed of three focus groups. However, the third focus group could not be conducted because many participants called in sick or were otherwise unavailable. While the sample size was small, participant comments generally echoed similar concerns and suggestions, and rural residents of Hawaii share similar care coordination-related issues as other rural areas including long travel distance to care, higher proportions of residents with lower socioeconomic status, and limited access to specialty oncology services. Lastly, the focus group interviews were all conducted virtually to allow participants to join remotely, due to the pandemic and to avoid the need for inter-island travel. This could mean limited information about nonverbal cues such as body language and eye contact that may have been more discernible from a traditional in-person interview.

Despite certain limitations, our study is significant because there are limited prior studies about how specific CC processes impact rural cancer care. Furthermore, this is the first focus group study regarding CC conducted with the rural Hawaii population. Hawaii is often excluded in US population-based studies, and this study provides insights into unique challenges with cancer care coordination for rural Hawaii patients. This study can help inform areas of improvements for cancer CC needed to help decrease disparities and increase survival in rural cancer patients.

## Conclusions

Our findings explored the challenges in rural Hawaii cancer patients’ CC experiences. Access to care presented as a major challenge due to the lack of oncologist and oncology services, limited local general medical providers, and the fragmented care system. Other challenges that influenced the cancer CC experience include travel issues and insurance-related troubles. We also identified facilitators of CC that are likely to provide positive CC experiences, including the use of electronic patient portals, team-based approach to care, support from family caregivers, and the presence of local clinic staff. Overall, our findings highlight potential targets of interventions to improve cancer care delivery for rural patients.

### Electronic supplementary material

Below is the link to the electronic supplementary material.

Supplementary Material 1

## Data Availability

The data collected and analyzed during this study are available from the corresponding author on reasonable request. Any information made available will be fully de-identified.

## Abbreviations

RUCA: Rural-Urban Commuting Area Codes
CC: Care Coordination
PCPs: Primary Care Providers
