# Supplementary material for: Cancer care coordination in rural Hawaii: a focus group study
Source: BMC Health Serv Res. 2024 Apr 24;24:518. doi: 10.1186/s12913-024-10916-1 (PMC11043031; doi:10.1186/s12913-024-10916-1)
Supplement: Supplementary file 1 — Supplementary Material 1 [file 12913_2024_10916_MOESM1_ESM.docx]

**Rural Care Coordination Study Focus Group Guide**

Purpose of FG

- The purpose of this focus group is to learn more about your experience with cancer care coordination on the neighbor islands----
- We hope to learn more about your experiences with cancer care so that we can improve care coordination for all cancer patients.
  - - Care coordination includes things like: communication among healthcare providers, communication between you and the people providing your healthcare, ease and availability of scheduling appointments, and access to the people providing your oncology care when you might have questions or need support. (Similar to many of the questions you answered in the survey)
- Please feel free to share your point of view, and there are no right or wrong answers in this discussion.

***Main Discussion***

- Thinking about your experience with care coordination for your cancer treatment, how was the care coordination process like for you?
- What was most helpful for you in coordinating your care?
- What aspects of care coordination were not helpful?
- If anyone had traveled to Oahu or the mainland for treatment, how was your care coordination experience for outer island treatment?
- The care coordination survey that you had completed was from within the last year, during the COVID pandemic.
  - How and what extent, if any, do you feel your care was impacted by the pandemic?
- On the survey, some participants had indicated that it took about 1-2 month to start cancer treatment.
  - How was your first treatment visit scheduled and coordinated?
  - If you experienced delays in starting treatment, what led to the delays in your treatment?
- If you have a primary care provider, how is your primary care provider involved in your cancer care?
- Our research team is interested in learning about what are some of the ways to improve care coordination in rural areas.
  - We would like to know:
    - what are some of your advice or suggestions about how to improve care coordination specifically on the neighbor islands?

Wrap up

- *Include a brief summary of discussion here.*
- Is there anything that we missed? Is there anything that you might have wanted to say that you didn't get a chance to say?

***Conclusion***

The End!

Thank you so much for coming and sharing your thoughts with us. We couldn’t do this study without you. We will email you your Amazon e-gift card in the next 1-2 weeks. If you would like us to email or call you once the results of the study are available, please let us know and we will send you the summary of what we find in the study when it is completed.
